# Practical *N*-Hydroxyphthalimide-Mediated Oxidation of Sulfonamides to *N*-Sulfonylimines

**DOI:** 10.3390/molecules24203771

**Published:** 2019-10-19

**Authors:** Jian Wang, Wen-Jing Yi

**Affiliations:** 1 Sichuan Industrial Institute of Antibiotics, Chengdu University, Chengdu 610052, China; 2 College of Chemistry and Environmental Protection Engineering, Southwest Minzu University, Chengdu 610041, China; ywjchem@163.com

**Keywords:** *N*-hydroxyphthalimide, nitroxides, oxidation, sulfonylimine

## Abstract

A new method to prepare sulfonylimines through the oxidation of sulfonamides mediated by *N*-hydroxyphthalimide under mild conditions has been developed. Compared to reported oxidation methods, broader substrates scope and milder conditions were achieved in our method. Importantly, this oxidation method can afford *N*-sulfonyl enaminones using Mannich products as starting materials. Additionally, the one-pot Friedel–Crafts arylation reaction of unseparated *N*-sulfonylimine formed in our system with 1,3,5-trimethoxybenzene was successful without any additional catalyst.

## 1. Introduction

**Figure 1:**
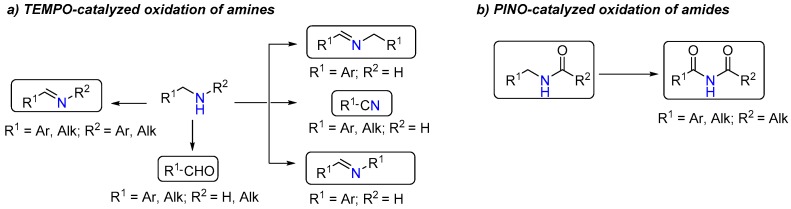
2,2,6,6-Tetramethylpiperidine-1-oxyl (TEMPO) and phthalimide *N*-oxyl (PINO) involved oxidation of amine-containing substrates.

Because of their low environmental impact, nitroxides are widely used in organic synthesis as carbon-centered radical scavengers, mediators of redox processes, and primary oxidants for transition metal catalyzed processes [1,2,3]. Both stable and short-living nitroxides are well studied as catalysts in various reactions [4,5,6,7,8,9,10]. An example of commercially available stable nitroxide is 2,2,6,6-tetramethylpiperidine-1-oxyl (TEMPO), and most of its reaction mechanisms involve the in situ generated oxoammonium species [11]. Among the short-living nitroxides, phthalimide *N*-oxyl (PINO) derived from *N*-hydroxyphthalimide (NHPI) is a prominent example. Highly reactive PINO usually triggers reactions by hydrogen abstraction or electron abstraction [12]. Base on the different mechanisms, TEMPO and PINO have been applied to activate different types of reaction substrates. TEMPO catalyst systems have been mainly limited to the oxidation of alcohols [13], while PINO catalyst systems are mostly focused on various aerobic oxidations of aliphatic and alkylaromatic hydrocarbons [14]. As counterparts of alcohols, amine-containing substrates are less widely explored by utilizing TEMPO or PINO catalyst systems. Although some reactions about TEMPO-catalyzed oxidation of primary and secondary amines have been reported, amides are usually not included (Figure 1a). One exceptional and distinguished research work reported by Togo’s group presented an oxidation of sulfonamides to *N*-sulfonylimines catalyzed by TEMPO [15]. However, this reaction condition was relatively complex and time-consuming. The imines products were sensitive to water, especially under this basic condition. On the other hand, due to the degradation of the NHPI by amine, only protected amine was studied as a reactant in PINO systems (Figure 1b) [16,17]. In conclusion, developing new nitroxides catalyst systems for the oxidation of amine-containing substrates is becoming more interesting.

**Figure 2:**
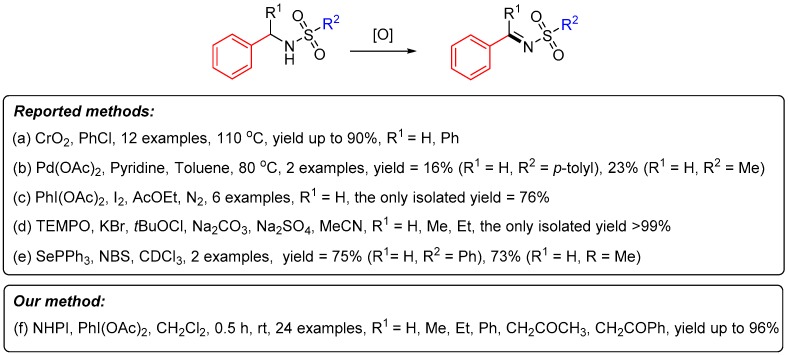
Oxidation conditions from sulfonamides to *N*-sulfonylimines.

*N*-Sulfonylimines are one of the most important imines due to their versatility in organic synthesis [18,19]. The traditional synthetic method for *N*-sulfonylimines, based on sulfonylimina- tion of aldehydes, usually needs elevated temperatures or strong acids due to the intrinsic reversibility of this condensation reaction [20,21,22,23,24]. Dean–Stark apparatus or molecular sieves are needed to remove the by-product H_2_O during these methods. It is not the best choice to use this method to synthesize *N*-sulfonylimines, especially when the structures are not stable in strong acid. What’s more, it is known that the condensation between ketones and sulfonamides to form *N*-sulfonylimines, especially enolizable ketimines, is usually more difficult [25]. In contrast, oxidation of sulfonamides to *N*-sulfonylimines would circumvent this problem under mild conditions. However, not too many procedures have been reported about the oxidation of sulfonamides to *N*-sulfonylimines (Figure 2) [15,26,27,28,29]. Some disadvantages of them, such as the use of metal catalysts, high temperatures and being air- or water-sensitive, still need to be addressed by other new oxidation methods. Higher yield and larger substrate scope should also be pursued. As aforementioned, a mild and environmentally benign nitroxide catalyst system has shown potential in the oxidation of amides. Here we demonstrate the first oxidation reaction of sulfonamides using a mild and practical metal-free NHPI mediator system.

## 2. Results and Discussion

Our initial investigations were focused on the transition-metal-free oxidation of *N*-benzyl-toluenesulfonamide (**1aa**) to *N*-sulfonylimine (**2aa**) using TEMPO as catalyst, and the results are summarized in Table 1. We knew that the oxoammonium species was the real active oxidizing agent in TEMPO catalyst systems. 4-Acetamido-2,2,6,6-tetramethylpiperidine-1-oxoammonium tetrafluoroborate (Bobbitt’s Salt) is one famous commercially available oxoammonium salt. In order to prove the feasibility of TEMPO working as a catalyst in our reaction, we first used a stoichiometric amount of Bobbitt’s Salt to directly react with **1aa** (Table 1, entry 1). A 32% yield under this condition showed oxoammonium salt was effective for this oxidation. It may be that one appropriate oxoammonium salt generated in situ from TEMPO could afford higher yield. Therefore, conditions where oxoammonium salt could be generated in situ from TEMPO by consuming secondary oxidants were tried. Unfortunately, no sufficiently high yield was obtained under catalytic conditions utilizing the interconversion between TEMPO and corresponding oxoammonium salt. When increasing the amount of TEMPO and oxidants largely, Selectfluor/TEMPO systems can give acceptable yields (Table 1, entry 3). However, this condition is defective due to the use of superstoichiometric amounts of expensive Selectfluor. Therefore, we anticipated that replacing TEMPO with short-living nitroxides such as PINO would achieve a more economical method. We were pleased to find that a 93% yield of **2aa** was obtained in half an hour by using a reported oxidative system in which the PINO radical could be generated in situ from NHPI (Table 1, entry 4) [30,31]. A 0.5 equivalent of NHPI was needed to mediate this oxidation reaction smoothly, and 0.2 equivalent NHPI afforded just a 34% yield of product (Table 1, entry 5). Dichloromethane (DCM) was decided as the best solvent for this reaction after several control experiments (Table 1, entry 6–8). When tetrahydrofuran (THF) was used as solvent, almost no product was detected. It is easy to understand that the reason is the α position to the oxygen atom of THF is active enough to terminate the propagation of radical intermediates during this reaction progress [32]. Several other user-friendly oxidants were tried, and only trichloroisocyanuric acid (TCCA) and *N*-Bromosuccinimide (NBS) could generate products with a pretty low yield (Table 1, entry 9–15). Both TCCA and *N*-Chlorosuccinimide (NCS) would give *N*-chlorinated amides as major product, especially in the case of NCS [33,34]. At last, the necessity of NHPI and PhI(OAc)_2_ was investigated by control experiment. When NHPI or PhI(OAc)_2_ was absent in the optimal conditions, start materials remained intact (Table 1, entry 16 and 17). Analogues of NHPI, such as 1-hydroxybenzotriazole (HOBT) and *N*-hydroxy succinimide (HOSU), were also able to promote this reaction, but their yields all decreased in different degrees (Table 1, entry 18 and 19).

**Figure 3:**
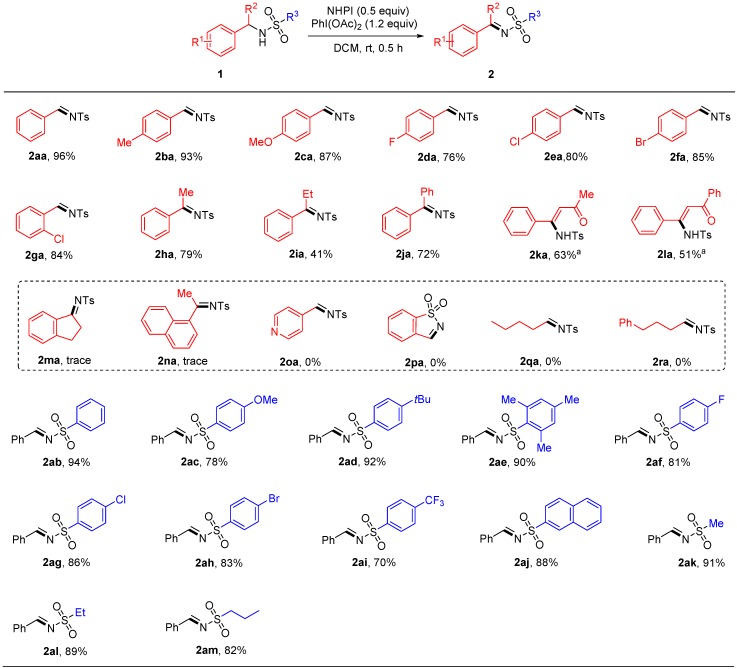
NHPI-mediated oxidation of sulfonamides to *N*-sulfonylimines. Reaction conditions: **1** (0.5 mmol), NHPI (0.25 mmol), PhI(OAc)_2_ (0.6 mmol), DCM (1.0 mL), rt, 0.5 h. [a] 1.0 equiv. NHPI, 2.0 equiv. PhI(OAc)_2_.

With the optimized reaction conditions in hand (Table 1, entry 6), the substrate scope of this transformation was further investigated (Figure 3). A variety of substituted sulfonamides were examined. We first investigated the substituents at the aromatic ring of benzylamine moiety. When 4-methyl-*N*-(4-methylbenzyl)benzenesulfonamide (**1ba**) was used in this reaction, high yield was obtained for the corresponding product **2ba**. One strong electron-donating group (4-OMe) could also be tolerated, and an 87% yield was obtained (**2ca**). With benzylamine subunits substituted by electron-withdrawing groups, such as F, Cl and Br, the sulfonamides still reacted smoothly and delivered high yields of products (**2da**–**2ga**). Next, reactant molecules in which R^2^ were other bulky substituent groups were tried; when R^2^ was the methyl or phenyl group, the yield of reaction remained relatively high despite a decrease compared with reactions where R^2^ was hydrogen (**2ha**, **2ja**). When R^2^ was ethyl, an obvious reduction of yield down to 41% occurred, and half of the starting materials **1ia** could be recycled. In order to make a complete conversion of **1ia**, more equivalents of NHPI and PhI(OAc)_2_, compared to standard conditions, were used. Although more **1ia** can be consumed by this way, fewer product **2ia** were obtained. Next, different types of substrates such as **1ma**-**1ra** were tried. Unfortunately, they afforded just trace products or did not react at all even at a higher temperature (60 °C), and left only a large amount of starting materials. Significantly, two Mannich products in which R^2^ was MeCOCH_2_ and PhCOCH_2,_ that could be easily prepared according to one three-component direct Mannich reaction between aldehydes, *p*-toluenesulfonamide, and enolizable ketones, was also tried [35]. Moderate yields of *N*-sulfonyl enaminones which were probably generated from the isomerization of sulfonylimines can be achieved with 1.0 equivalent NHPI and 2.0 equivalent PhI(OAc)_2_ (**2ka**, **2la**). It should be noted that the preparation of *N*-sulfonyl enaminones has not been well developed, especially the inherent drawback of the condensation method encountered using asymmetric diketones and sulfamides [36,37]. Our oxidation method can forge the desired double bond directly. Next, different sulfonyl groups containing aromatic and aliphatic substructures were researched. In general, all the sulfonyl derivatives acquired high yield (**2ab**–**2am**) except **2ai**, ranging from 78% to 92%. In the case of **2ai**, the decrease of yield suggested that the strong electron-withdrawing group in this part was undesirable in this oxidation.

**Figure 4:**
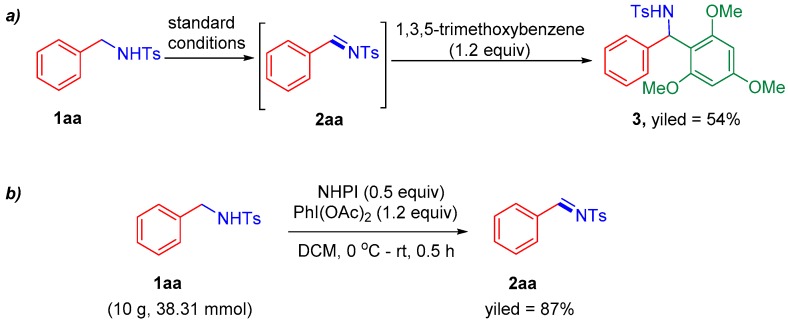
One-pot Friedel–Crafts arylation of sulfonylimine and Gram-Scale reaction.

To demonstrate the applications of this practical oxidation protocol, we continued to study the subsequent additional reactions to *N*-sulfonylimines formed in the oxidation reactions without further purification. Therefore, one-pot Friedel–Crafts arylation reactions of sulfonylimines with electron-rich arenes were tried according to the reported literature [38]. To our delight, when 1,3,5-trimethoxybenzene was used as the nucleophile for this designed arylation reaction, a moderate overall yield of 54% of two steps was obtained (Figure 4a). This experiment indicates our reaction system is potentially capable of addition reactions to *N*-sulfonylimines, and unstable sulfonylimines which are hard to isolate can be utilized through in-situ preparation by our method. In addition, the large-scale reaction using our method was also successful after a minor adjustment of reaction conditions (Figure 4b). The oxidant PhI(OAc)_2_ must be added carefully at a low temperature portion-wise, otherwise, the drastic exothermicity may cause an explosion.

**Figure 5:**
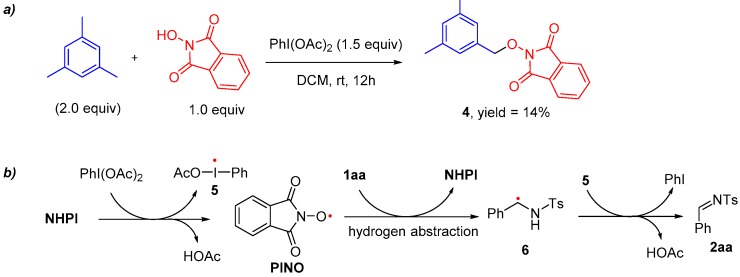
Radical scavenging experiment and proposed mechanism.

According to the literature, it has been demonstrated that PhI(OAc)_2_ was able to initiate the generation of PINO radical from NHPI [30,31]. It is also known that the PINO radical was easy to abstract a hydrogen atom from a benzylic position of the organic substrate [39,40,41]. We designed one radical scavenging experiment (Figure 5a) to validate that the PINO radical was indeed formed in our reaction system. Product **4** in this experiment was formed by selective radical/radical cross-coupling between the PINO radical and the benzylic radical which came from hydrogen abstraction of mesitylene, and this explanation is supported by reported literature [40]. On the basis of the above observation and other literature reports [42,43,44], we propose a mechanism where the key step is the hydrogen abstraction reaction from starting materials by the PINO radical (Figure 5b). Then radical **5** oxidizes the α-sulfonamido carbon radical **6** to obtain the final product. Hence, the generation of radical **6** and the stability of **6** probably determine the yield of the overall reaction. However, it is hard to identify the specific reason why substrates **1ma**–**1ra** are unreactive during this oxidation system.

## 3. Experimental Sections

### 3.1. General

All reagents were purchased from Aldrich Chemical Co. (Darmstadt, Germany), Adamas-beta (Shanghai, China) and Energy Chemical (Shanghai, China) and used without further purification. All starting materials sulfonamides was synthesized following standard literature procedures [35,45,46]. Silica gel (Adamas-beta, Shanghai, China) column chromatography was carried out to purify products. Proton nuclear magnetic resonance (^1^H-NMR, 600 MHz) and carbon-13 nuclear magnetic resonance (^13^C-NMR, 150 MHz) spectra were measured on a JNM-ECZ600R/S1 (JEOL, Tokyo, Japan) with CD_3_CN or CDCl_3_ as solvent and recorded in ppm relative to an internal tetramethylsilane standard. High-resolution mass spectra (HRMS) were recorded on a 6520 Q-TOF MS system (Agilent, Santa Clara, CA, USA) using an electrospray (ESI) ionization source. Known compounds were confirmed by ^1^H-NMR spectra according to literatures.

### 3.2. Experimental Method

#### 3.2.1. General Procedure for NHPI-Mediated Oxidation of Sulfon-amides to *N*-Sulfonylimines

A dried reflux tube equipped with a magnetic stir bar charged with sulfonamides (0.5 mmol, 1.0 equiv.), NHPI (0.25 mmol, 0.5 equiv.) and DCM (1 mL), then PhI(OAc)_2_ (0.6 mmol, 1.2 equiv.) was added in one portion, the reaction mixture was stirred at room temperature for 0.5 h under air. The mixture was directly purified by flash column chromatography eluting with ethyl acetate and hexane to afford *N*-sulfonylimines.

#### 3.2.2. Procedure for Gram-Scale reaction

A dried reflux tube equipped with a magnetic stir bar charged with sulfonamides (10 g, 1.0 equiv.), NHPI (3.12 g, 0.5 equiv.), and DCM (50 mL) at 0 °C, then PhI(OAc)_2_ (14.8 g, 1.2 equiv.) was carefully added portion-wise over 10 min, and the reaction mixture was stirred at room temperature for 0.5 h under air. The mixture was directly purified by flash column chromatography eluting with ethyl acetate and hexane to afford *N*-sulfonylimines.

### 3.3. Characterization Data of New Compounds

*N-Benzylidene-4-(tert-butyl)benzenesulfonamide* (**2ad**). white solid; mp 121.0–123.0 °C; ^1^H NMR (600 MHz, CD_3_CN) δ 9.06 (s, 1H), 7.94 (d, *J* = 7.4 Hz, 2H), 7.89 (d, *J* = 8.4 Hz, 2H), 7.68–7.63 (m, 3H), 7.53 (t, *J* = 7.8 Hz, 2H), 1.31 (s, 9H); ^13^C NMR (150 MHz, CD_3_CN) δ 172.1, 158.7, 136.1, 136.0, 133.4, 132.0, 130.0, 128.6, 127.4, 35.9, 31.1; HRMS (ESI): [M + H]^+^ calcd. for C_17_H_20_NO_2_S: *m*/*z* = 302.1209; found, 302.1213.

*N-Benzylidene-4-fluorobenzenesulfonamide* (**2af**). white solid; mp 69.0–71.0 °C; ^1^H NMR (600 MHz, CD_3_CN) δ 9.04 (s, 1H), 8.03–8.00 (m, 2H), 7.94 (d, *J* = 7.8 Hz, 2H), 7.66 (t, *J* = 7.2 Hz, 1H), 7.52 (t, *J* = 7.5 Hz, 2H), 7.32 (t, *J* = 8.8 Hz, 2H); ^13^C NMR (150 MHz, CD_3_CN) δ 172.5, 136.1, 133.2, 132.1, 131.9, 131.8, 130.2, 117.6, 117.4; HRMS (ESI): [M + H]^+^ calcd. for C_13_H_11_FNO_2_S: *m*/*z* = 264.0489; found, 264.0493.

*N-Benzylideneethanesulfonamide* (**2al**). colorless oil; ^1^H NMR (600 MHz, CD_3_CN) δ 9.03 (s, 1H), 8.01 (d, *J* = 6.7 Hz, 2H), 7.73–7.67 (m, 1H), 7.61–7.52 (m, 2H), 3.24–3.20 (m, 2H), 1.35–1.30 (m, 3H); ^13^C NMR (150 MHz, CD_3_CN) δ 173.7, 135.9, 133.5, 132.0, 130.2, 47.5, 8.1; HRMS (ESI): [M + H]^+^ calcd. for C_9_H_12_NO_2_S: *m*/*z* = 198.0583; found, 198.0584.

*N-Benzylidenepropane-1-sulfonamide* (**2am**). colorless oil; ^1^H NMR (600 MHz, CD_3_CN) δ 9.02 (s, 1H), 8.01 (dd, *J* = 8.2, 1.2 Hz, 2H), 7.72-7.68 (m, 1H), 7.58 (t, *J* = 7.8 Hz, 2H), 3.20–3.16 (m, 2H), 1.86–1.77 (m, 2H), 1.03 (t, *J* = 7.4 Hz, 3H); ^13^C NMR (150 MHz, CD_3_CN) δ 173.4, 135.9, 133.5, 132.0, 130.2, 54.6, 17.7, 13.1; HRMS (ESI): [M + H]^+^ calcd. for C_10_H_14_NO_2_S: *m*/*z* = 212.0740; found, 212.0741.

^1^H and ^13^C NMR spectra of these compounds are available in the Supplementary Materials.

## 4. Conclusions

In summary, we have developed a new and mild oxidation method where sulfonylimines or *N*-sulfonyl enaminones can be easily prepared. Our method is capable of achieving some multisubstituted sulfonylimines with a moderated yield, which have needed relatively harsh conditions when using the traditional condensation methods between ketones and sulfonamides. Further studies on developing more types of substrates and synthetic applications of this oxidation reaction are currently underway.

## Figures and Tables

**Table 1:**
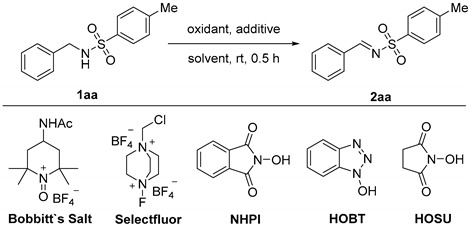
Optimization of reaction conditions ^a^.

Entry	Oxidant (equiv)	Additive (equiv)	Solvent (mL)	Yield (%) ^b^
1	Bobbitt’s Salt (2.0)	-	CH_3_CN (2)	32
2	PhI(OAc)_2_ (2.0)	TEMPO (2.0)	CH_3_CN (2)	30
3	Selectfluor (2.0)	TEMPO (2.0)	CH_3_CN (2)	79
4	PhI(OAc)_2_ (1.2)	NHPI (0.5)	CH_3_CN (1)	93
5	PhI(OAc)_2_ (1.2)	NHPI (0.2)	CH_3_CN (1)	34
6	PhI(OAc)_2_ (1.2)	NHPI (0.5)	DCM (1)	96
7	PhI(OAc)_2_ (1.2)	NHPI (0.5)	EtOAc (1)	81
8	PhI(OAc)_2_ (1.2)	NHPI (0.5)	THF (1)	trace
9	TCCA (1.2)	NHPI (0.5)	DCM (1)	42
10	NBS (1.2)	NHPI (0.5)	DCM (1)	37
11	NCS (1.2)	NHPI (0.5)	DCM (1)	trace
12	MCPBA (1.2)	NHPI (0.5)	DCM (1)	0
13	BPO (1.2)	NHPI (0.5)	DCM (1)	0
14	K_2_S_2_O_8_ (1.2)	NHPI (0.5)	DCM (1)	0
15	TBHP (1.2)	NHPI (0.5)	DCM (1)	0
16	PhI(OAc)_2_ (1.2)	-	DCM (1)	0
17	-	NHPI (0.5)	DCM (1)	0
18	PhI(OAc)_2_ (1.2)	HOBT (0.5)	DCM (1)	54
19	PhI(OAc)_2_ (1.2)	HOSU (0.5)	DCM (1)	85

^a^ Reaction conditions: **1aa** (0.5 mmol), additive and solvent was mixed, then oxidant was added, rt, 0.5 h; ^b^ Isolated yield.

## Supplementary Materials

The following are available online, ^1^H NMR, ^13^C NMR spectra of products.
