# Supplementary material for: Practical N-Hydroxyphthalimide-Mediated Oxidation of Sulfonamides to N-Sulfonylimines
Source: Molecules. 2019 Oct 19;24(20):3771. doi: 10.3390/molecules24203771 (PMC6832120; doi:10.3390/molecules24203771)
Supplement: Supplementary file 1 [file molecules-24-03771-s001.pdf]

Practical *N*-Hydroxyphthalimide-Mediated Oxidation of Sulfonamides to *N*-Sulfonylimines

Jian Wang [a, *], Wen-Jing Yi [b]

[a] *Sichuan Industrial Institute of Antibiotics, Chengdu University, Chengdu, 610052, P. R. China*

[b] *College of Chemistry and Environmental Protection Engineering, Southwest Minzu University, Chengdu, 610041, P. R. China*

General information:

^{1}H-NMR spectra were measured on a JEOL-600M with CD_3CN as solvent and recorded in ppm relative to internal tetramethylsilane standard.

Copies of new compounds ^{1}H NMR and ^{13}C NMR

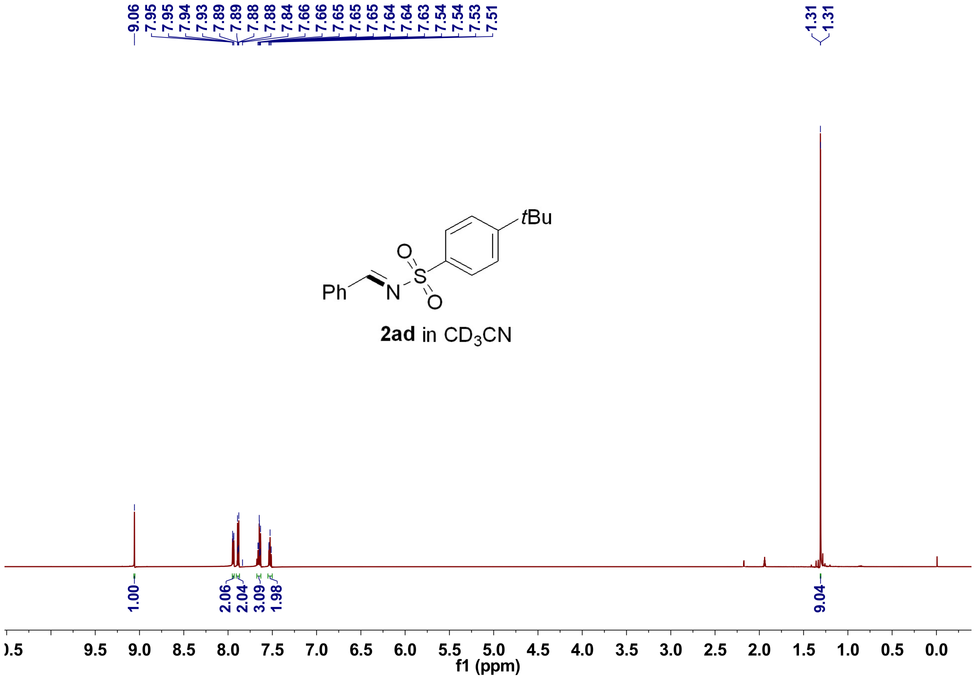

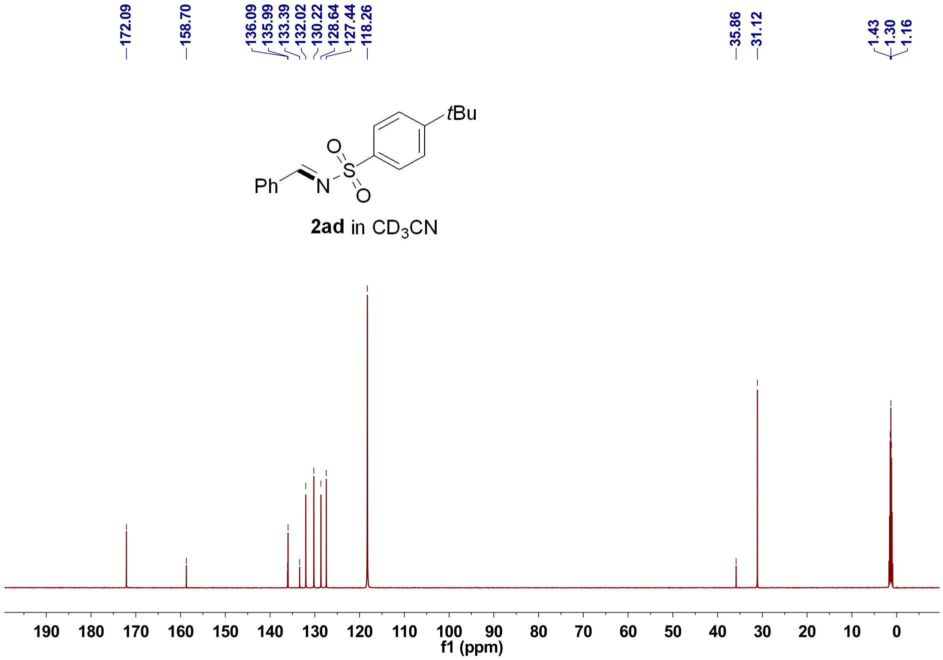

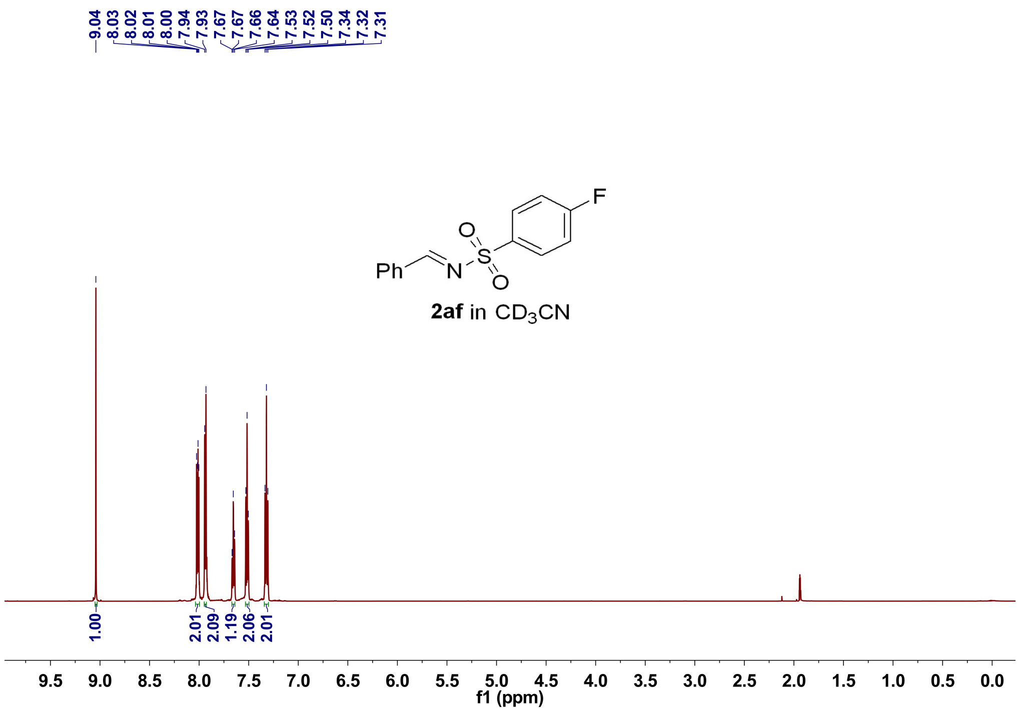
9.04
8.03
8.02
8.01
8.00
7.94
7.93
7.67
7.67
7.66
7.64
7.53
7.52
7.50
7.34
7.32
7.31
F
O
S
Ph
N
O
2af in CD_3CN
1.00
2.01
2.09
1.19
2.06
2.01
9.5 9.0 8.5 8.0 7.5 7.0 6.5 6.0 5.5 5.0 4.5 4.0 3.5 3.0 2.5 2.0 1.5 1.0 0.5 0.0
f1 (ppm)

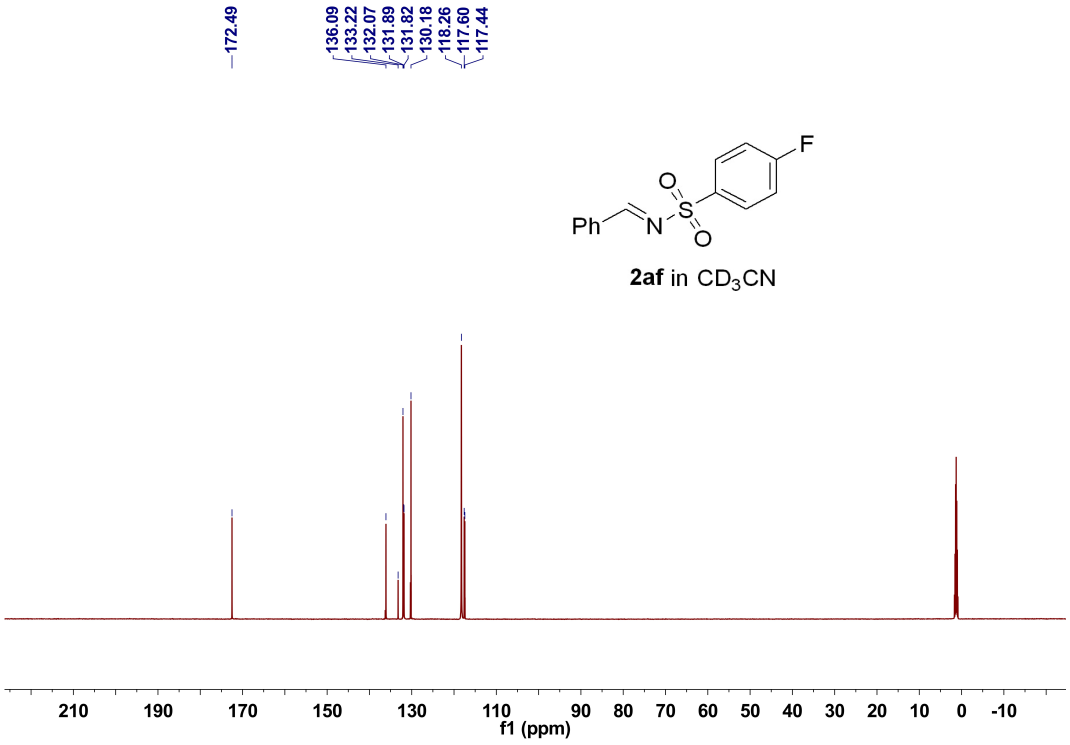
172.49
136.09
133.22
132.07
131.89
131.82
130.18
118.26
117.60
117.44
F
O
S
Ph
N
O
2af in CD_3CN
210 190 170 150 130 110 90 80 70 60 50 40 30 20 10 0 -10
f1 (ppm)

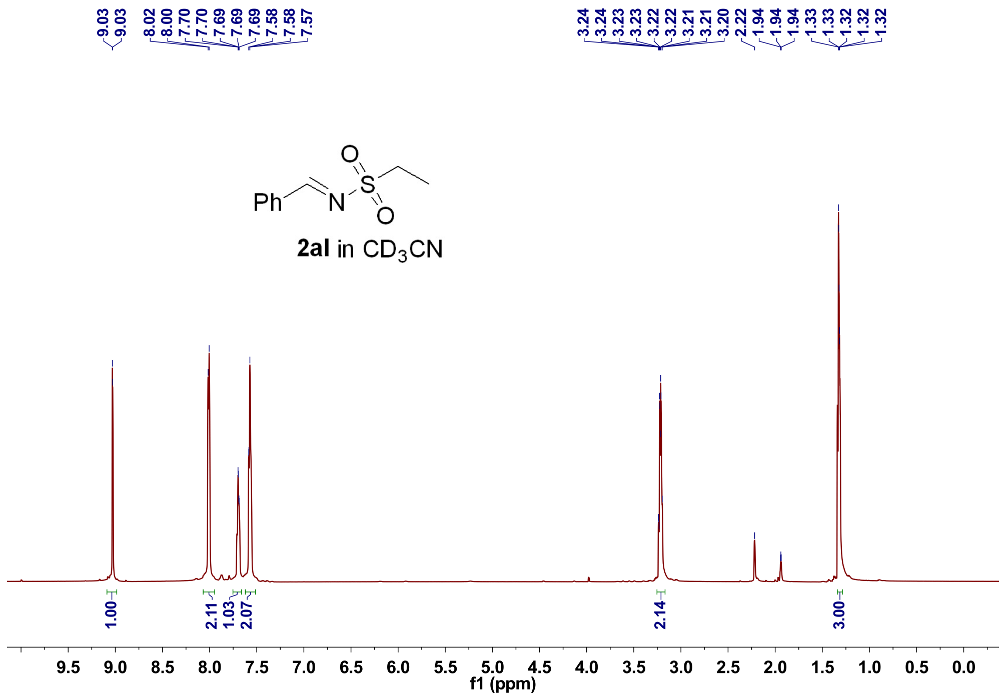

9.03
9.03
8.02
8.00
7.70
7.70
7.69
7.69
7.69
7.58
7.58
7.57
3.24
3.24
3.23
3.23
3.22
3.22
3.22
3.21
3.21
3.20
2.22
1.94
1.94
1.94
1.33
1.33
1.32
1.32
1.32
Ph
N
S
O
O
2al in CD_3CN
1.00
2.11
1.03
2.07
2.14
3.00
9.5 9.0 8.5 8.0 7.5 7.0 6.5 6.0 5.5 5.0 4.5 4.0 3.5 3.0 2.5 2.0 1.5 1.0 0.5 0.0
f1 (ppm)

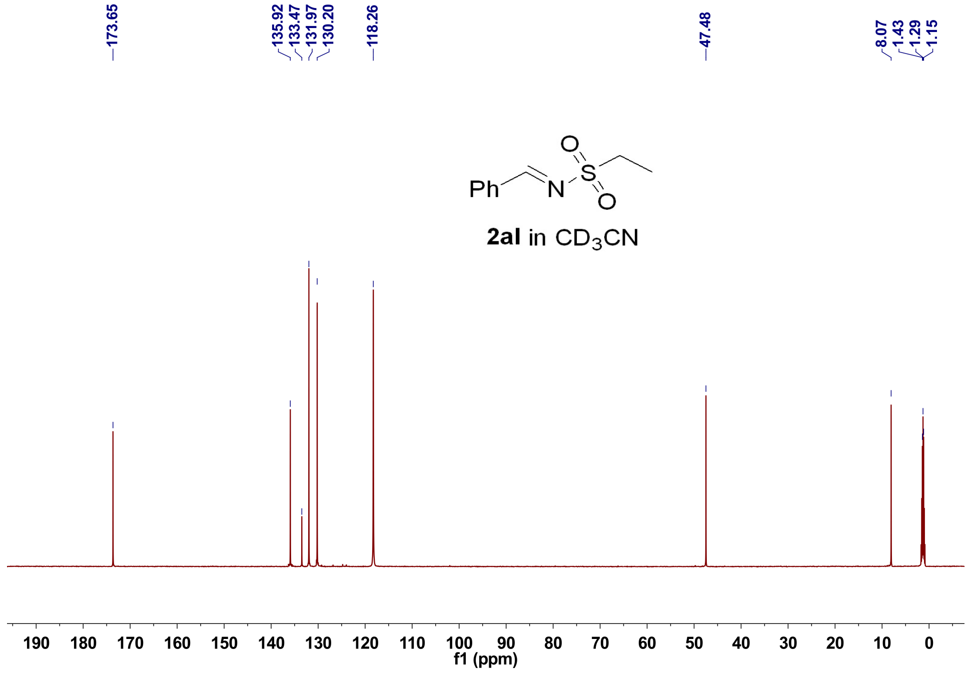

173.65
135.92
133.47
131.97
130.20
118.26
47.48
8.07
1.43
1.29
1.15
Ph
N
S
O
O
2al in CD_3CN
190 180 170 160 150 140 130 120 110 100 90 80 70 60 50 40 30 20 10 0
f1 (ppm)

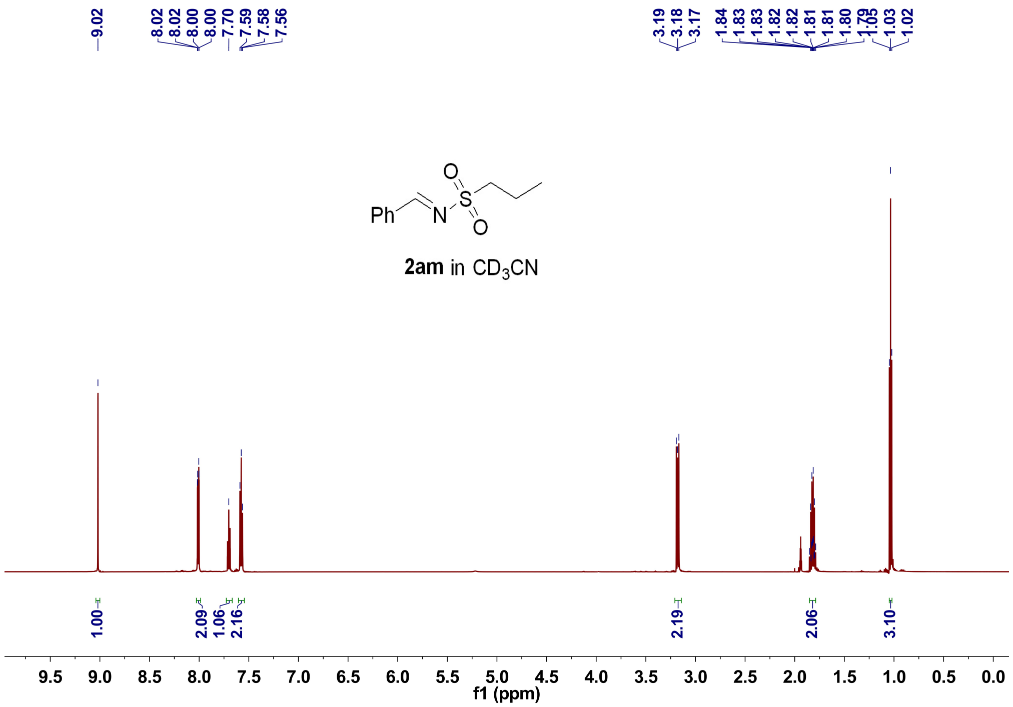
9.02
8.02
8.02
8.00
8.00
7.70
7.59
7.58
7.56
3.19
3.18
3.17
1.84
1.83
1.83
1.82
1.82
1.81
1.81
1.80
1.79
1.05
1.03
1.02
Ph
N
S
O
O
2am in CD_3CN
1.00
2.09
1.06
2.16
2.19
2.06
3.10
9.5 9.0 8.5 8.0 7.5 7.0 6.5 6.0 5.5 5.0 4.5 4.0 3.5 3.0 2.5 2.0 1.5 1.0 0.5 0.0
f1 (ppm)

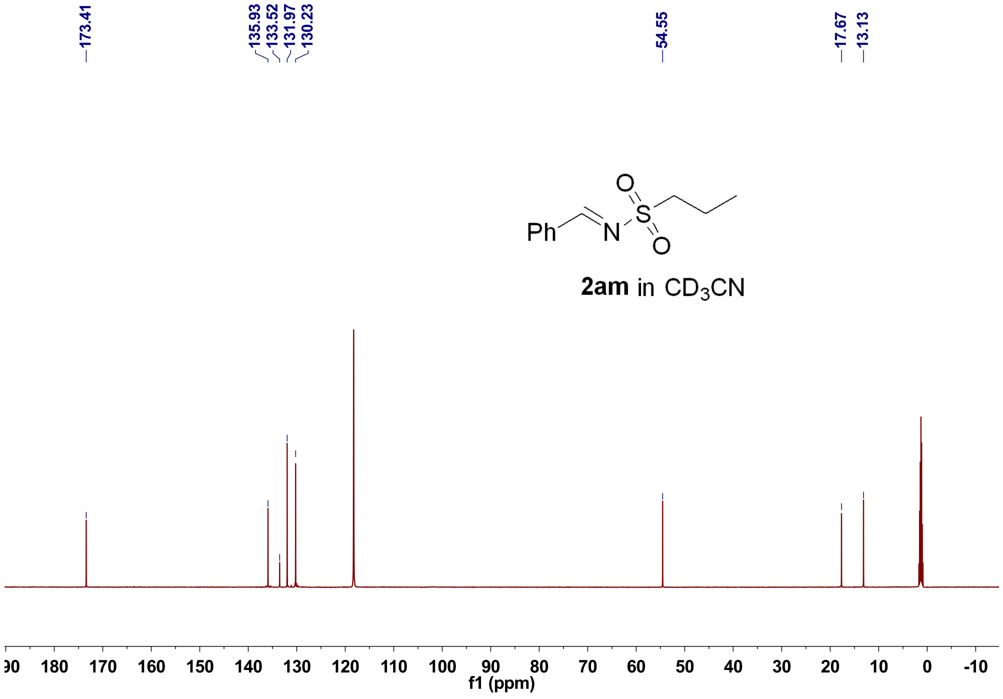
173.41
135.93
133.52
131.97
130.23
54.55
17.67
13.13
Ph
N
S
O
O
2am in CD_3CN
180 170 160 150 140 130 120 110 100 90 80 70 60 50 40 30 20 10 0 -10
f1 (ppm)
